# Fighting *Fusarium* Pathogens in the Era of Climate Change: A Conceptual Approach

**DOI:** 10.3390/pathogens9060419

**Published:** 2020-05-28

**Authors:** Salme Timmusk, Eviatar Nevo, Fantaye Ayele, Steffen Noe, Ülo Niinemets

**Affiliations:** 1 Department of Forest Mycology and Plant Pathology, Swedish University of Agricultural Sciences, P.O. Box 7026, 75007 Uppsala, Sweden; stimmusk@yahoo.com; 2 Bashan Institute of Science, 1730 Post Oak Ct, Auburn, AL 36830, USA; 3 International Graduate Centre of Evolution, University of Haifa, Haifa 3498838, Israel; nevo@research.org; 4 National Academy of Sciences, Washington, DC 20418, USA; 5 Ethiopian Biotechnology Institute, Addis Ababa 60002, Ethiopia; 6 Chair of Crop Science and Plant Biology, Estonian University of Life Sciences, 51006 Tartu, Estonia; noe@emu.ee (S.N.); ylo.niinemets@emu.ee (Ü.N.); 7 Estonian Academy of Sciences, 10131 Tallinn, Estonia

**Keywords:** ecosystem–atmosphere relations, plant microbiome, *Fusarium*, bacterial exopolysaccharides, genomic networks, sustainable development

## Abstract

Fusarium head blight (FHB) caused by *Fusarium* pathogens is one of the most devastating fungal diseases of small grain cereals worldwide, substantially reducing yield quality and food safety. Its severity is increasing due to the climate change caused by weather fluctuations. Intensive research on FHB control methods has been initiated more than a decade ago. Since then, the environment has been rapidly changing at regional to global scales due to increasing anthropogenic emissions enhanced fertilizer application and substantial changes in land use. It is known that environmental factors affect both the pathogen virulence as well as plant resistance mechanisms. Changes in CO_2_ concentration, temperature, and water availability can have positive, neutral, or negative effects on pathogen spread depending on the environmental optima of the pathosystem. Hence, there is a need for studies of plant–pathogen interactions in current and future environmental context. Long-term monitoring data are needed in order to understand the complex nature of plants and its microbiome interactions. We suggest an holobiotic approach, integrating plant phyllosphere microbiome research on the ecological background. This will enable the development of efficient strategies based on ecological know-how to fight *Fusarium* pathogens and maintain sustainable agricultural systems.

## 1. Introduction

Climate change has resulted in significant changes in weather patterns, precipitation distribution, and temperature fluctuations (FAO 2008). Extreme conditions caused by these changes bring about many unexpected and more frequent biotic and abiotic stresses [1,2,3,4], in particular, novel combinations of stress severities [5]. At the same time the average annual variation in environmental factors might not change dramatically. To feed an increasing world population, total crop production needs to be significantly increased, but this becomes increasingly difficult due to lack of fertile arable land and more severe biotic and environmental stress conditions [6,7,8,9,10,11].

*Fusarium* (*Hypocreales*, *Nectriaceae*) is one of the most renowned genera in the *Fungi* kingdom. It includes, in a broad sense, a large number of morphologically and phylogenetically diverse fungi, commonly found as air-, soil-, or water-borne saprobic organisms, but also found in dead or living plant material as endophytes or epiphytes [12]. Many *Fusarium* spp. are effective plant pathogens or secondary invaders of worldwide concern. While numerous species are mycotoxigenic and cause devastating human and animal diseases, they often occur isolated from their immunocompromised hosts [12]. Based on the perceived scientific and economic importance, *Fusarium* spp. were recently included in the top 10 globally most important genera of plant pathogenic fungi [13]. This, in particular, reflects the economic importance of the diseases caused by *F. graminearum* [13].

Fusarium head blight (FHB) is one of the most devastating fungal diseases worldwide. FHB is caused by a complex of up to 20 different co-existing *Fusarium* species [14,15,16,17,18], substantially reducing cereal yield and quality and negatively impacting food safety. The outbreaks of the disease occur especially in years with warm and humid weather conditions resulting in yield losses of up to 75% [19]. Furthermore, the severity of *Fusarium* infections is expected to further increase in future warmer climates [18]. In addition to yield loss the other major concern is the change in seed quality [17,20]. Contamination of grain with mycotoxins leads to a quality loss for human consumption and poses difficulties in processing and feeding contaminated grain to animal species [14,15,16,17,18,21]. The most frequently encountered *Fusarium* mycotoxins in Europe are type B trichothecenes (TCTB). The family of TCTB mycotoxins include deoxynivalenol (DON) and its acetylated forms 15-acetyl-4-deoxynivalenol and 3-acetyl-4-deoxynivalenol (15-ADON and 3-ADON), and nivalenol (NIV) and its acetylated form fusarenone X (FX) [14,15,16,19,22]. TCTB mycotoxins have different acute and chronic effects on humans and animals and they were reported to be the primary cause of alimentary toxic aleukia, which has caused death of thousands of people in the USSR during the 1940s [23]. Currently, the biggest concern with TCTB mycotoxins does not result from short-term exposure to high mycotoxin concentrations, but from prolonged exposures that lead to chronic health effects [14,15,16,17,19,23]. Therefore, maximum allowable DON levels in food products have been established http://www.fao.org/3/y5499e/y5499e07.htm and EC regulation EC856/2005 amended in July 2007/EC number 1126/2007) [23].

## 2. FHB Counteracting and Preventive Measures

In order to reduce the FHB risks and grain TCTB mycotoxin accumulation, several control practices have been shown to be efficient. These control strategies rely on plant resistance breeding and on reducing the levels of primary pathogen inocula through management strategies like crop rotation, tillage, and the use of fungicides. Breaking the fungal disease cycle by adapting the sowing period [19,24] has proven to be a good measure too. However, the period when spraying of fungicides is effective might be very short and, depending on appropriate weather conditions, difficult to handle [19,25]. Additionally, the efficacy of the fungicide application determines if all *Fusarium* species which are present and pathogenic under particular conditions can be controlled. Sometimes the application of a fungicide which works excellently against some specific pathogenic species might not be effective against other species, which subsequently become dominant [19,21]. These factors of inefficient treatment lead to variable success in controlling FHB by application of fungicides [19,21].

Plant FHB resistance breeding is an extremely complex task because multiple genes control the disease quantitative traits. Additionally, the resistance breeding outcome depends on the genotype and environmental interactions [19,26,27,28,29,30]. Early studies on FHB resistance have demonstrated that the resistance can be broken down into two components: type I resistance that operates against initial infection, and type II resistance that operates against the spread of the pathogen within the host [28,29,30]. Later, three additional components were differentiated: a resistance to kernel infection (type III), a tolerance to infection (type IV), and a resistance to DON accumulation (type V) [28,29,30]. Over 100 quantitative trait loci (QTL) for FHB resistance in wheat (*Triticum aestivum*) have been identified so far [26,29,30]. QTL for FHB disease linked with the heading date, plant height, spikelet morphology, kernel discoloration were found in all wheat chromosomes except chromosome 7D [26,28,29,30,31]. Wheatgrass *Thinopyrum elongatum* is an important wild relative of wheat with many excellent resistance traits and can be an effective source for FHB resistance breeding. The perennial plant was sequenced, and chromosomal locations of genes along with genetic markers identified. After multiple crosses and silencing the candidate genes individually it was found that one, Fhb7 codes for an enzyme, glutathione S-transferase, which is required for wheat FHB resistance [31].

It is clear that environmental change will impact not only the crop plant–pathogen interactions but also other aspects of the plant cropping system that ultimately will affect the *Fusarium* inoculation potential and host plant susceptibility [22]. The economic severity of the disease depends on the disease geographic distribution, climatic conditions, and crop susceptibility [32]. Recently, changes in FHB population composition dynamics and disease virulence have been recorded [33], suggesting that the knowledge of FHB resistance needs to be updated. Furthermore, a detailed understanding of the climatic controls on pathosystem is required for the development of effective management strategies [33].

## 3. Biocontrol

The EU directive 2009/128 requests a strengthening of the integrated pest management (IPM). That means precise application and overall reduction of the usage of pesticides to minimize their impact on human health and environment [34]. As the primary *Fusarium* pathogen inoculum source is crop residues, a sound approach would be the application of biocontrol agents to the soil in order to reduce the survival of the pathogen [18,19,25]. Application of biocontrol agents (BA) is hampered by a lack of knowledge of the conditions under which *Fusarium* species become dominant. For example, if *F. graminearum* is suppressed by the application of antagonistic bacteria that are used as BA and/or the application of a species-specific fungicide, still large quantities of other *Fusarium* species might occur, especially, if they had previously been suppressed by *F. graminearum* [19,25]. Several microbial strains from *Clonostachys Trichoderma Bacillus, Pseudomonas*, and *Paenibacillus* genera have been identified as acting antagonistically to *F. graminearum* under lab and field [19,35,36,37,38] but, no commercial BA is yet available. An appropriate strategy for discovery and development of BA can only be generated thorough understanding the active principles and mechanism of their action [39,40,41]. We established a progressive screening method for FHB biocontrol agents: plate assay combined with a wheat kernel assay system which allows robust screening of high number of *biocontrol* isolates [39,42]. Generally the BA bioactive lipopeptide antibiotics production has been considered as mechanism of biocontrol [42]. In the case of an efficient biocontrol agent *Paenibacillus polymyxa* A26, initial plate assays with wild type and its Sfp-typePPTase mutant confirmed that its lipopeptide products have critical importance for the strain antagonistic ability [42]. Studies with the more complex system employing wheat kernels show, however, that the BA-enhanced exopolysaccharides (EPS) production may be of major importance in antagonizing the pathogen in case the lipopeptide antibiotics are not produced. Hence *P. polymyxa* A26 biofilm EPS compounds along with the bacterially produced lipopeptides are capable of antagonizing *F. graminearum*. These results were confirmed in greenhouse experiments [39] and simultaneous qPCR monitoring of A26, and pathogens combined with mycotoxin assay supported this finding [42]. Furthermore, it was further shown that the EPS uronate content is of critical importance for the biocontrol efficiency [39]. The system has been currently used to monitor and optimize the BA formulation strategies. To maintain the BAs active principles during storage is one of the challenges with biocontrol strains. Therefore it is quite important to have a simple two component system for routine screening of stored biocontrol agents [42]. 

## 4. The Phyllosphere

Plants harbor different species- and cultivar-specific microbial communities that grow as epiphytes and endophytes in the rhizosphere and phyllosphere [43]. The phyllosphere microbiome is composed of various microorganisms but bacteria are the most abundant cellular organisms in the phyllosphere community, and the most efficient colonizers are present in numbers between 10^6^ and 10^7^ cells cm^−2^ of leaf tissue [43,44,45,46]. The bacteria in phyllosphere are often vertically transmitted as endophytes and they form patchy assemblages on plant surface called biofilms [39,47,48,49] or microbial consortia [39,45,46,50]. While the rhizosphere and endo-rhizosphere have been thoroughly studied for centuries, less is known about the drivers that influence the phyllosphere, i.e., any aerial part of the plant, such as stems, leaves, leaf ears, or flowers [50,51]. This is a significant limitation as phyllosphere microbiome is of critical importance for dispersal of *Fusarium* pathogens. Although *Fusarium* spp. can survive several years on plant debris, even if located deep in the soil, the pathogens themselves, however, develop only on the upper parts of living plants [20]. Hence, the review focuses on the phyllosphere, which has a large potential for the design of beneficial microbial biofilm layers [52,53].

The phyllosphere is a highly nutrient-limited habitat compared to the rhizosphere [50,51,54]. Phyllosphere is characterized by permanently changing temperature and humidity regimes, whereas the phyllospheric environmental conditions are predicted to become aggravated in warming climates [50,51,54]. However, in cooler climates such as boreal and temperate biomes, current global warming patterns generally stimulate biofilm formation primarily through the increased winter survival [3]. Thus, the expected changes in cool biomes can lead to a higher phyllosphere biofilm formation [50,51,54].

**Figure 1:**
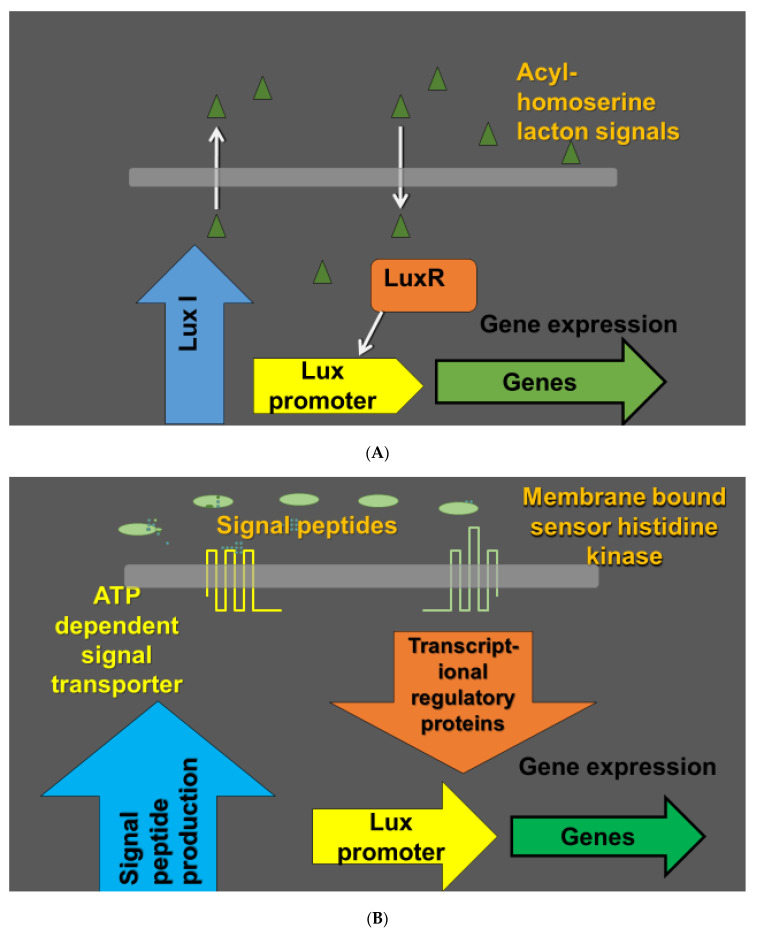
Quorum sensing in bacteria as a cell to cell signaling mechanism. Populations of bacteria can operate in a coordinated manner depending on environmental conditions and the density of the bacterial population. The coordination of the bacterial population performance is based on secretion of signaling molecules called autoinducers. Bacterial cells detect the autoinducers and coordinate the regulation of expression of particular genes in dependence of the autoinducer concentrations. In Gram negative bacteria, the autoinducers are typically acyl-homoserine lactones (acyl-HSL). Lux I family enzymes (Lux I-type acyl-HSL synthases) catalyze the formation of species-specific homoserine lactones. Acyl-HSL are detected by lux R type transcription regulators (**A**). Gram positive bacteria use 8–10 amino acid long short oligopeptides and membrane bound sensor histidine kinases as receptors. As the membrane is not permeable to the peptides, specialized transporters mediate secretion of the quorum sensing peptides. The oligopeptide signaling is mediated by DNA binding transcription regulatory proteins (**B**) [62].

**Figure 2:**
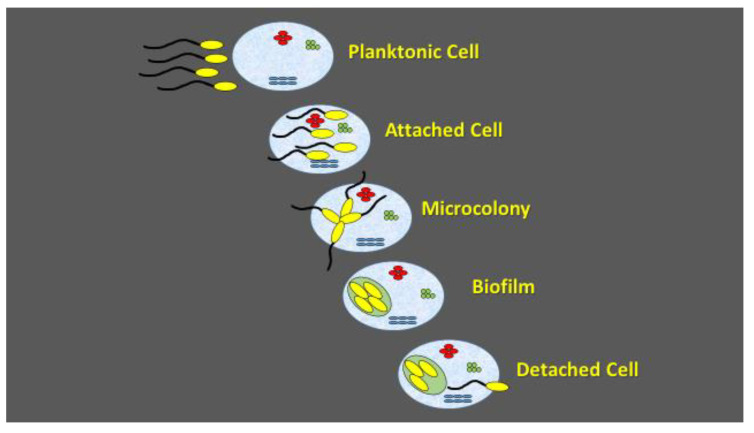
Schematic presentation of biofilm formation.

Biofilms are considered as microniches which allow the bacteria to operate as a functional unit and enable them to accomplish tasks that are not possible at their planktonic stage. Biofilms consist of cells and matrices where complex exopolysaccharides (EPS) and proteins are major components [55,56,57,58]. Biofilm environment provides an important means for survival of phyllospheric microbia in natural ecosystems [55,56,57,58]. Several studies have revealed the role of quorum sensing in biofilm formation [59,60,61] where microbial cells use chemical signals for coordination prior to biofilm formation. Quorum sensing permits the microbes to determine the environmental conditions of the actual population [59,61,62]. N-acyl-homoserine lactones together with oligopeptide autoinducers are the main diffusible signals employed in bacterial communication (Figure 1) [59,61,62]. Biofilm formation is therefore a dynamic balanced process to promote planktonic cells attachment until microcolony formation allowing cell migration and new colony formation (Figure 2) [59,61,62]. Apart from quorum sensing, the microorganisms communicate with each other, with the plant, and other organisms through emission of volatile organic compounds (VOC) [63]. VOCs are of great importance as signaling molecules and in the plant chemical defense by either directly suppressing pathogens [64] or serving as volatile cues eliciting defense responses in neighboring not yet infected leaves [65,66].

Biological control in the phyllosphere can be accomplished through direct antagonism via production of antibiotics or via the induction of plant resistance [67]. The advantage of biological control pathways that involve the plant own immunity is that these defense mechanisms can stay active even after the infecting agent is no longer present and therefore can potentially provide a longer-term benefit [23]. A large set of plant compounds, that are activated by phyllospheric microorganisms can initiate transcriptional reprogramming of the infected plant tissue [23,61]. Competitive exclusion of pathogens by the broader phyllosphere community may play an important role in plant pathogen resistance [39].

As already mentioned, along with lipopeptide antibiotics the bacterial biofilm EPS with high uronate content are capable of antagonizing *F. graminearum* [39,42]. Microorganisms in their natural environments are subject to various fluctuations of environmental conditions that can be altered by the EPS matrix that serves as the microbial interface with the environment. It has been suggested that the ecological ‘benefit’ provided by EPS within the biofilm depends on the potential to favorably influence the acclimation of microorganisms to the environment [68]. In addition to environmental adaptation, EPS are also known to promote communication, compartmentalization, competence, and defense [69]. As the production of EPS requires large amounts of energy, their regulatory control is of vital importance and the regulation of EPS synthesis occurs at many levels. Different subpopulations of genes encoding EPS are activated during different stages of biofilm development [70]. Several EPS molecules have a high water holding capacity (WHC), up to 15 times their mass [39,40,42,52,69,71,72,73]. Such a high water accumulation potential can protect against drought stress and has been suggested as a mechanism of drought tolerance enhancement strains [39,40,42,52,69,71,72,73,74]. Considering the presence of bacterial natural phyllosphere EPS there is a huge potential for novel biocontrol agent discovery. Even though the EPS layer is dependent upon the perception of numerous environmental signals from the host and the ecosystem, this would open the new range of application of EPS in integrated pathogen management.

Biofilm formation is an environmental microbiology concept, denoting microbial cell adaptation to multicellular lifestyle. The temporal sequence of events in biofilm formation starts with planktonic cell attachment, followed by microcolony formation and cell detachment. Biofilm formation maximizes nutrient delivery to all viable cells and increases resistance to environmental stress factors. The attachment is initiated by exuding extracellular polysaccharide material that entraps cells and debris within a glue-like matrix. During the biofilm development, the bacterial cells are self-immobilized in the extracellular polymeric matrix that is capable of supporting rapid growth of the microorganisms and the microcolonies are formed. As the microcolonies become established and their thickness increases, mature biofilms are formed. Quorum sensing is used as the signal for planktonic cell attachment, microcolony formation, as well as for cell dispersal [75].

## 5. Concept of Plant Health

**Figure 3:**
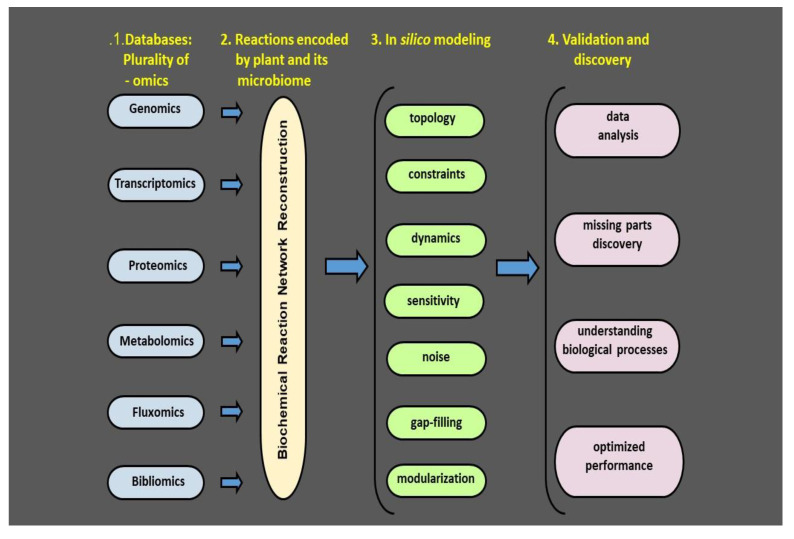
Plant and its phyllosphere microbiome: a four-step model for metabolic systems biology.

Despite importance of plant heath for forestry and agriculture its meaning is not fully conceptually developed [76]. The reductionist approach is rooted in investigating small parts of pathosystems. Interactions with pathogens as well as beneficial microbes have been studied for years in different academic fields. The reductionist approach is rooted in investigating small parts of pathosystem, and the broad scientific field is currently highly fragmented. In particular, interactions of plants with pathogens and with beneficial microbes have been studied for years in different academic fields. Microbial community interaction networks have been studied by microbial ecologists, volatiles by chemical ecologists, environmental signals by climate and soil scientists, and interactions with harmful microorganisms are in the domain of plant pathologists. As a result, over the years, each of the fields has developed its own approach and language [77]. It is clear that our current challenge is to strengthen the connections between ecological and agricultural studies [77,78]. Thanks to the methodological progress over the last decades a great deal of the complexity of plant–microbe interactions can be currently investigated (Figure 3 and [53]). By combining multi-omics technology, high resolution immunological detection methodology and molecular genetics, new insights into plant microbe interactions can be provided [77,78]. Complex metabolic processes can be converted into a mathematical framework of the underlying biochemical, genetic and genomic controls, allowing the formulation of genomic scale models. These models enable computation of the phenotypic traits of a system of interest. The number of genomic network reconstructions has grown exponentially during recent years [53,79]. We can now estimate numerous cellular processes including elicitation of VOC release and describe their interactions chemically and mathematically, and as a result, identify the environmental constraints under which the network operates. This makes it possible to optimize the physiological functions of a given phyllospheric biofilm in coping with environmental challenges like droughts. We argue that interdisciplinary approaches based on reliable mechanistic frameworks are needed to formulate microbial metabolic genotype–phenotype connections which can be included into multi-model ensembles predicting the microbial responses to different environmental challenges [80]. Likewise, we suggest ecosystem level studies with different crops and disease combinations. Such studies will serve as an input for integration of different-scale models from biochemistry to microbial community, ultimately enabling process-based understanding of performance of microbial communities [41].

Recent advances in multiomic techniques have made it possible to examine the microbial communities under natural conditions. Genomics, transcriptomics proteomics, metabolomics, fluxomics, and bibliomics provide information on genes, their expression, protein products, and their specific activities. This information together with literature databases makes it feasible to reconstruct the biochemical reaction network. The network forms a basis for in silico modeling followed by validation in relevant biological systems, ultimately providing an overview of the phyllosphere microbial isolates and their bioactive compounds. Having this information at hand, we will be able to create a map of the crop plant microbiome and microbiome metabolic network. An effective monitoring method should detect plant stress and/or its alleviation in the first stages before the stress severity is detected by the visual symptoms. Hence a plant microbiome map is a map of risk in terms of probability of introduced microbiome colonization, fate, and efficiency. The aim of such a map is to find an optimum strategy to maximize the isolate’s expected positive effect and minimizing the time it takes. Adapted from *Bacillus thuringiensis* AZP2 metabolic system [53].

## 6. Atmospheric Context

It is clear that both pathogen virulence mechanisms as well as plant resistance pathways are affected by environmental factors [81]. Changes in CO_2_ concentration, temperature, and water availability can have positive, neutral, or negative effects depending on the environmental optima of the pathosystem considered [81]. As a general practice, most of the plant–pathogen interaction research has been performed under a few static conditions that do not allow capturing the pathosystem dynamics as it occurs in the nature. Hence, there is a need to include environmental studies with sufficient temporal resolution in order to understand the complex nature of plant–pathogen interactions [81]. There are multiple advantages in treating the phyllosphere biofilm communities independently from rhizopheric communities. As an example, UV light has a high impact on microbes exposed to it on phyllospheric biofilms, but plays no role for rhizospheric biofilms. Using state-of-the-art methodology like high sensitivity sensors for environmental characteristics enables the study of fundamental mechanisms of microbiological community organization and their composition in relation to apparent changes in the aerial environment. First, simple comparisons among phyllospheric microbiomes can be made across plant species, varieties, and treatments. Given that sometimes the counteracting measures act independently, we should continue to analyze each community independently in every experiment. We should, however, consider that in natural settings plants are exposed to an enormous variety of complex, ever-changing atmospheric signals and variations in environmental drivers such as light, temperature, and humidity [82]. These signals may help or hinder the biofilm formation and modulate its performance over time and therefore we need to constantly monitor the environmental drivers and the biofilm reactions. We should assume that several components of the biofilms are acting in an interdependent manner, and treating them as independent can be a source of misinterpretation. The general practice in plant phenotyping is that the phenotype is defined based on statistical analysis including principal component analysis (PCA), multivariate and cluster analysis techniques that help to identify the most reproducible components and eliminates components with a weak influence [83]. However, this can lead to a biased choice, especially in cases where each of the multiple environmental variables lead to weak phenotypic reactions in the plant–pathogen system and are therefore not chosen to be principal components. For example, a change in a process that has a small effect on several biofilm components might not have a detectable effect on any single biofilm component. Strategies to avoid such biases depend on techniques where appropriate pools of genotypes or phenotypes are formed and by that boost the signal-to-noise ratio to allow effective detection of multiple component, which can ultimately improve the detection of the remaining signal for each component [53,77].

**Figure 4:**
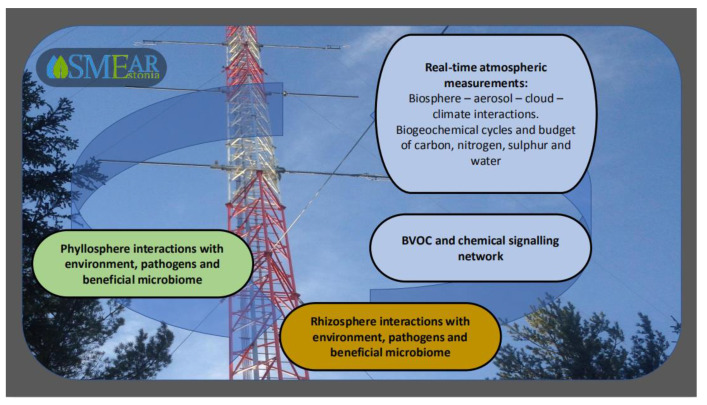
The station for measuring ecosystem–atmosphere relations (SMEAR) Estonia concept in tracing the environment and plant interactions.

Hence, the biofilm community application studies have to be performed against a background that integrates state-of-the-art observations at various scales. Long-term, comprehensive, and integrated plant phyllosphere and atmosphere measurements are of key importance for understanding the dynamics and feedback mechanisms between the plant surface colonizers and their environment. Such observations are in progress in Estonia at the station for measuring ecosystem–atmosphere relations (SMEAR Estonia) (Figure 4) [82,84]. At SMEAR Estonia, comprehensive long-term real-time measurements of meteorological characteristics and atmospheric trace gases, atmospheric particulate matter, and air ions are conducted together with ecophysiological measurements at plant and soil levels to obtain data of individual plant growth, gas-exchange characteristics and phyllosphere/root/soil variables. These measurements are combined with continuous monitoring of ecosystem net primary productivity. The most important trace gases measured are carbon dioxide, methane, ozone, nitrogen oxides, and sulphur dioxide. In addition, anthropogenic (typically primary organic aerosols such as soot) and natural atmospheric aerosol (secondary organic aerosols, SOA) particles are measured at the site. Natural new particle formation in forest atmospheres occurs as the result of condensation of trace gases of organic compounds onto organic or inorganic molecules. Besides SOA, forest atmosphere includes biological material such as pollen grains and bacterial, algal, and fungal spores [85,86]. Aerosols affect the atmospheric radiation balance and can thus importantly affect plant physiological activity and life on plant surface. For instance, aerosols increase the share of diffuse radiation in total solar radiation, enhancing plant growth and ecosystem gross primary productivity (GPP) [84,87]. On the other hand, deposition of particulate matter onto trees and crops have been reported to reduce growth, partly due to covering plant surfaces and hindering the gas-exchange with the atmosphere, but also due to interaction with gaseous pollutants [88,89]. Especially in urban atmospheres, gaseous pollutants are typically present at high levels in atmospheres enriched with particulate matter [88,89,90], and can significantly reduce plant physiological activity [91,92,93] and change the conditions for organisms colonizing the aerial surface [94,95,96].

The multidisciplinary and multiscale approach covers processes in spatial dimensions ranging from nanometers to square kilometers, being thus able to significantly contribute to worldwide measurement networks that can ultimately contribute to understanding vegetation–climate interactions, including the controls of environmental drivers on plant productivity and abiotic and biotic stress, and effects of plant productivity on environment through reduction of atmospheric CO_2_ and production of biogenic trace gases and aerosols [81,84,87]. In addition, this integrated measurement network allows estimation of medium to long-distance dispersal of beneficial and pathogenic phyllosphere microbial components, thus providing a basis to study the connectivity of different phyllosphere communities and their modification under changing climates [84].

The SMEAR Estonia is a comprehensive large-scale scientific infrastructure that provides continuously high-quality data of a large set of environmental parameters [97]. Utilizing high end measurement equipment and a long-term dataset enables the design of plant–microbial interaction experiments under natural environmental conditions. 

## 7. Concluding Remarks

We propose to re-integrate plant pathology with microbial community science, agronomy, ecology, and atmospheric sciences in order to understand the dynamics of the plant phyllosphere and its interactions with the environment. Comprehensive and continuous observations of a complex network of factors influencing pathogen virulence and spread are needed to enable mitigating strategies. Here we presented a design for combined observation networks to monitor temporal changes in environment, phyllosphere microbial consortia, and *Fusarium* dispersal. Integrating the information of plant species- and variety-specific phyllosphere microbiome with the ecological background will enable development of efficient strategies that integrate the ecological know-how to fight *Fusarium* pathogens in the context of global change and maintain sustainable agricultural systems.
